# Perceptions of the health impacts of climate change among Canadians

**DOI:** 10.1186/s12889-023-15105-z

**Published:** 2023-01-31

**Authors:** Nora Casson, Laura Cameron, Ian Mauro, Karl Friesen-Hughes, Rhéa Rocque

**Affiliations:** Prairie Climate Centre, University of Winnipeg, 515 Portage Ave, R3B 2E9 Winnipeg, MB Canada

**Keywords:** Climate change, Health impacts of climate change, Risk perceptions, Climate opinions, Canada

## Abstract

**Background:**

Understanding public perceptions of the health risks of climate change is critical to inform risk communication and support the adoption of adaptive behaviours. In Canada, very few studies have explored public understandings and perceptions of climate impacts on health. The objective of this study was to address this gap by exploring perceptions of the link between climate change and health.

**Methods:**

We conducted a survey of Canadians (n = 3,014) to address this objective. The 116-question survey measured prior consideration of the link between climate change and health, affective assessment of climate health impacts, unprompted knowledge of climate health impacts, and concern about a range of impacts. ANOVA tests were used to assess differences among sociodemographic groups.

**Results:**

Overall, Canadian’s have a similar level of concern about health impacts of climate change compared with concern about other impacts (e.g. biophysical, economic, and national security). Among health-related impacts, respondents were more concerned about impacts on water, food and air quality, compared with impacts on mental health, infectious diseases and heat-related illnesses. There were differences among sociodemographic groups; women were significantly more concerned than men about all of the health-related impacts; respondents with a high school level of education were significantly less concerned about all health-related impacts compared with respondents with more education; and respondents on the political left were more concerned with those in the political centre, who were more concerned than those on the political right.

**Conclusion:**

There is emerging literature suggesting that framing communication around climate change in terms of the health risks it poses may increase perceptions of the proximity of the risks. These results suggest that it is important to be specific in the types of health risks that are communicated, and to consider the concerns of the target sociodemographic groups. The differential knowledge, awareness, and concern of climate health impacts across segments of the Canadian population can inform targeted communication and engagement to build broader support for adaptation and mitigation measures.

**Supplementary Information:**

The online version contains supplementary material available at 10.1186/s12889-023-15105-z.

## Introduction

From increased frequency and intensity of heat waves and wildfires to the proliferation of infectious diseases, food and water insecurity, and mental health challenges, climate change presents an immense threat to public health globally [1]. Health impacts of climate change are increasing, in intensity and frequency [2, 3], but the public are perhaps less aware of these risks compared with biophysical impacts of climate change [4]. Understanding public perceptions of the health impacts of climate change is critical for risk communication and ultimately the adoption of behaviours that support climate adaptation and mitigation [5].

Studies that have explored public perceptions of health and climate change have often found a relatively superficial understanding. A global systematic review of public and health professionals’ perceptions of the health implications of climate change found ten studies from English-speaking nations, and eight studies from non-English speaking nations on public perceptions of the health impacts of climate change [6]. The review found that the majority of North Americans perceive climate change as having negative health impacts, yet few can list specific issues or name who is specifically at risk without being prompted [6]. In a national US survey, Maibach et al. [4] found that “most Americans have little understanding of the health relevance of climate change.” Comparing surveys from the US, Canada, and Malta, Akerlof et al. [7] found that despite the majority of people across all three countries viewing climate change as a health risk, “climate change appears to lack salience as a health issue” based on the limited depth of answers to open-ended questions. Studies in other parts of the world such as Nepal [8], Bangladesh [9], and Hong Kong [10] have found high levels of awareness of climate change, but varying degrees of awareness around public health impacts depending on factors such as education and income.

In Canada, recent reports have summarized the latest research on the health impacts of climate change (e.g. [11, 12]), but very few studies have explored public understanding and perceptions of these impacts. A 2008 survey by Health Canada found that respondents did not often associate climate change with health impacts, but when prompted they were likely to accept their relationship [13, 14]. A subsequent national survey found a slight increase in Canadians’ knowledge of the health risks posed by climate change, though a minority of people actually took steps to protect themselves [15]. An interview-based study in southern Ontario found that 77% of participants identified health impacts of global environmental change when prompted, but were not able to describe this link in detail or in relation to their own lives [16]. A focus group case study exploring perceptions of climate change and Lyme disease in Manitoba also found low levels of understanding regarding the link between health and climate as well as the challenges and opportunities this presents for effective climate and risk communication [17].

Understanding public risk perception is not only important as an indication of public willingness to support climate adaptation and mitigation measures [18, 19], but also to inform public risk communication approaches and frames. There is an emerging body of research in the climate communications field suggesting that by communicating the health risks of climate change and focusing on the relevance of these issues to personal and community health and well-being, there is greater potential to engage a wider audience [20–22]. However, other studies have found that framing climate communications from a health perspective may not be a straightforward or universal approach, and there is a need to better understand how this framing may be operationalized [17, 23–25].

While Canada has an overall high level of awareness of climate change, there is substantial regional variation in attitudes [26], and the health impacts of climate change vary in type and severity across the country [17, 27, 28]. This makes Canada an interesting and valuable context in which to explore the nuances of framing climate communications from a health perspective. Perceptions of the risks of climate change depend on the proximity of those risks, either in space [29, 30]. or in time [31], and thus the demographic diversity and differences in health risks across the country make it possible to explore the extent to which this is important when using a health frame.

Given the urgency of communicating and acting on climate change combined with the evolving discourse regarding public health in the context of the COVID-19 pandemic, there is a need to understand Canadians’ perceptions of the health risks of climate change. To address this, we conducted a nationally-representative survey to explore public perceptions and awareness regarding the link between climate change and health in Canada. This study had three objectives: to determine perceptions of the link between climate change and health; to evaluate Canadians’ level of concern around specific health impacts of climate change; and to explore how climate and health risk perceptions can inform public engagement and communications. These results will help risk communication around climate-related health risks, and provide guidance about how to target this communication.

## Methods

### Study area

This study and associated survey sought to understand the perceptions and attitudes of Canadians regarding climate change and health. Framed by three oceans, Canada is the second largest country in the world by total area and has numerous large cities as well as diverse rural and remote regions. The survey was conducted across all ten provinces and three territories, among a total population of 36.99 million people (as of the 2021 Census).

### Development of survey instrument

The survey instrument was designed considering a review of surveys on climate change, health, and risk perceptions. Some questions were modified from previous studies [4, 13, 32, 33], while others were developed for the specific context of this study. The survey instrument was reviewed and pre-tested among the researchers and their networks and the final instrument contained 116 questions (the full survey instrument is in supplementary materials). Respondents were asked questions related to their perceptions, experiences, and prior consideration of climate change and health impacts as well as responses to specific communication materials (the latter of which will be reported in forthcoming publications). Most questions were asked on an 11-point scale, from zero to ten.

### Data collection

After approval by the University of Winnipeg Human Ethics Research Board, the survey was conducted from December 14–23, 2020, with support of Nanos Research. The survey was self-administered online by respondents in either English or French according to their preference. The sample was drawn from a probability panel, recruited through random digit. Research has found that online surveys from probability samples yield more accurate results than telephone interviews or nonprobability online surveys [34]. To ensure representation in less populated areas, recruitment was supplemented by phone calls which directed respondents to the online survey. Each section of the survey was on a separate webpage that did not allow for participants to review previous pages, and thus allowed more information to be revealed as the survey proceeded. Participants received a small compensation for their time of five dollars for completion of the survey. The response rate of the survey was 13%, perhaps attributed in part to the survey being distributed in December, close to the holidays and during the height of the second wave of COVID-19 across Canada. Studies on response rates within Canadian public health research demonstrate that online surveys have limitations (e.g. passive engagement, difficulty issuing reminders, etc.) that lead to the lowest response rates of all survey methods available [35]. However, the method was chosen in this case as the most cost effective and efficient to administer within a limited budget.

### Measurement of outcome variables and analysis

We measured sociodemographic variables and levels of concern related to climate change, health (including COVID-19), and other global issues through a range of questions in order to contextualize the overall concern about climate change of the population. To contextualize the concerns Canadians have about the health impacts of climate change, we asked survey respondents to rate their level of concern about climate change impacts in four categories: health impacts, biophysical impacts, economic impacts and impacts on national security (Q19 – Q34 in the survey). The data were normally distributed and had homogenous variances among subgroups, thus we used parametric tests (i.e. one-way ANOVA, two-way ANOVA and Tukey HSD tests). We used a two-way ANOVA and Tukey HSD tests to assess how levels of concern vary among these categories and across socio-demographic groups (age, gender, community size, politics, education, income). We assessed prior consideration of the link between climate change and health using one-way ANOVAs and Tukey HSD tests using Q35 as the response variable (*Before taking this survey, how much had you thought about how climate change might affect people’s health on a scale from 0 to 10, where 0 is hadn’t thought about it at all and 10 is thought about it a great deal?*). To understand Canadians’ perceptions of the link between climate change and health, we conducted one-way ANOVAs and Tukey HSD tests using Q36 as the response variable (*How would you rate the impact of climate change on people’s health on a scale of 0 to 10 where 0 is very good for people’s health and 10 is very bad for people’s health?*) and six socio-demographic variables as predictors (age, gender, community size, politics, education and income).

In order to identify knowledge of the health impacts of climate change when unprompted, respondents were asked the open-ended question: “In what ways, if any, do you think climate change will affect the health of Canadians?”. Three members of the researcher team developed a preliminary coding scheme, based on previous studies and literature on the health impacts of climate change relevant in Canada [15, 36]. Examples of initial code categories include air quality, temperature-related morbidity and mortality, and infectious diseases. A sample of the responses were reviewed independently by two researchers, and the initial coding scheme was added to and adapted. All responses were then coded through two rounds, conducted by one researcher in conversation with a second when discrepancies arose. A broad, holistic definition of the health impacts of climate change was adopted, including physical, mental and social aspects of health and well-being. Code frequencies were analysed following the approach of Baxter [37].

To assess health impacts of highest concern to Canadians, we asked respondents their levels of concern for 15 different health impacts of climate change (Q38 to Q52). These impacts fell into five categories: water and food borne diseases, air quality, mental health, temperature-impacts and infectious diseases. We used one-way ANOVAs and Tukey HSD tests to assess differences among and within categories of health impacts, and two-way ANOVAs to assess differences in concern about health impacts among categories and socio-demographic groups.

## Results

### Sample description

The sample includes 3,014 respondents, with sufficient sample sizes from each province and territory in Canada to allow for regional analyses (Table 1). The data used in the analysis were weighted by province, gender, and age so that the results reflect the demographic distribution of the Canadian population (2016 Census). Weighted and unweighted data are presented in Table 1, which shows the sociodemographic distribution of respondents. All figures and statistical analyses following Table 1 use weighted data.

**Table 1:** Sociodemographic characteristics of the sample

	N (unweighted) 3014	N (weighted) 3000	% (weighted)	Census (%)
**Gender**				
Female	1447	1544	51.5	50.9
Male	1567	1456	48.5	49.1
**Age (years)**				
18 to 34	477	820	27.3	19.5 ^†^
35–54	1198	1023	34.1	27.2
55+	1339	1157	38.6	30.9
**Province/Territory**				
*East*				
Newfoundland and Labrador	200	46	1.53	1.48
New Brunswick	200	65	2.17	2.13
Nova Scotia	200	81	2.70	2.68
Prince Edward Island	150	12	0.400	0.410
*Central*				
Quebec	451	702	23.4	23.2
Ontario	666	1148	38.3	38.2
*Prairies*				
Manitoba	200	105	3.50	3.64
Saskatchewan	200	90	3.00	3.13
Alberta	200	336	11.2	11.6
*West*				
British Columbia	397	406	13.5	13.2
*North*				
Territories	150	9	0.300	0.220

^†^ Census data is recorded in ages 15–19 and 20–24, so this value is scaled as a percentage of age 20 - 34

### Concern about climate change relative to other issues

The first set of questions on the survey asked respondents about their concern on several issues in order to contextualize their concern about climate change. Mean concern about climate change was 7.05 on the scale from 0 to 10 (standard deviation = 2.88). Climate change ranked third in the list of 9 concerns, significantly higher than race relations, national security, food security and foreign affairs, but significantly lower than the COVID-19 pandemic and the economy. Concern about climate change was not significantly different from concern about cyber attacks and concern about habitat and species decline based on the results of a one-way ANOVA (F = 37.53, p < 0.001).

### Health impacts of climate change are as concerning as biophysical and economic impacts

**Fig. 1:**
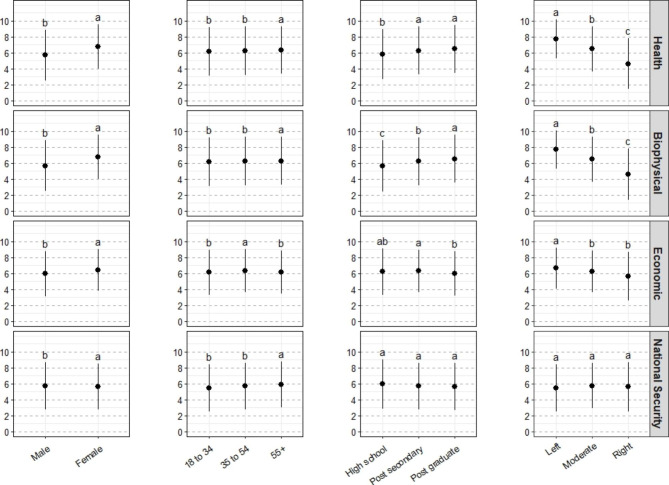
Concern about a range of climate impacts - grouped into the categories of national security, health, economic, and biophysical impacts - compared across four sociodemographic variables(weighted). Lowercase letters indicated statistical differences among sociodemographic groups

When asked their level of concern about a range of 16 climate impacts - within the broad categories of health, biophysical, economic, and national security impacts – respondents had significantly higher concern about health, biophysical and economic impacts compared with national security impacts (F = 21.39, p < 0.001) (Fig. 1; Table S1). There were significant effects of and interactions with each of the four sociodemographic categories. Female respondents had significantly higher concern than male respondents within each of the categories of impacts, and older respondents (55 +) had significantly higher concern compared with other age groups in terms of health, biophysical and national security impacts. The most striking demographic differences were found when comparing concern across levels of education and political leaning. For both biophysical and health impacts, the level of concern was significantly higher among respondents with more education and among respondents who identified as ‘left’ or ‘moderate’ on the political spectrum (Fig. 1). These demographic differences were less substantial for concern about economic impacts, and disappeared when asked about concern about national security impacts (Fig. 1).

### Prior consideration of the link between climate change and health varies across demographics

**Fig. 2:**
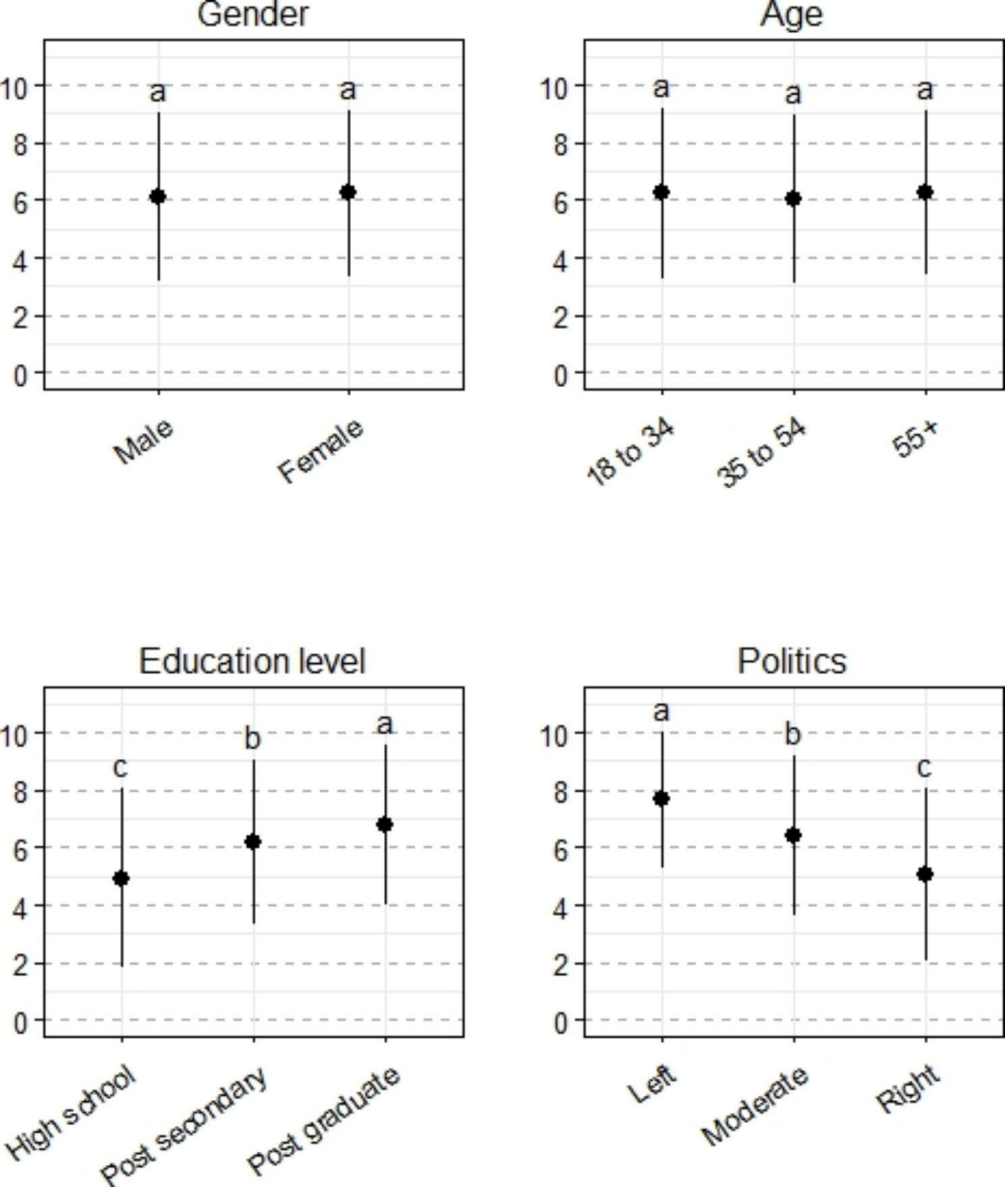
Prior consideration of the link between climate change and health across four sociodemographic variables. Lowercase letters indicate statistical differences among sociodemographic groups

When asked how much they had thought about the health impacts of climate change before taking this survey (i.e. prior consideration of the climate-health link), results show substantial consideration of this connection across the entire sample (mean 6.18, standard deviation = 2.90). There were no statistically significant differences in the consideration of the climate-health link among genders or age groups, but there were significant increases in consideration of the climate-health link as education level increased (F = 36.46, p < 0.001) and as political leaning shifted towards the ‘left’ (F = 99.55, p < 0.001) (Fig. 2).

There were significant, positive correlations between both concern about personal health and the consideration of the link between climate change and health (r = 0.22, p < 0.001), as well as between concern about COVID-19 and the consideration of the link between climate change and health (r = 0.21, p < 0.001).

### Most Canadians perceive climate change as bad for people’s health

On the affective assessment of climate change, with 0 being “very good for people’s health” and 10 being “very bad for people’s health”, the mean response was 7.15 (standard deviation = 2.29) across the whole sample. Women perceive climate change as worse for health compared with men (F = 113.32, p < 0.001), and there are also significant differences associated with education level (respondents with higher levels of education perceived climate change as significantly worse compare with lower levels of education, F = 17.44; p < 0.001), and political leaning (respondents who are politically left perceive greater negative health impacts from climate change compared with those in the centre, who also perceive the health impacts as worse than those on the right, F = 171.59; p < 0.001) (Figure S1).

### More than half of Canadians can name one or more health impact of climate change

In response to an open-ended question about the health impacts of climate change, 2162 respondents (of 3014 total respondents) wrote answers; 1740 answers were coded as including one or more health impacts, while 422 were not coded, as they did not answer the question. 540 respondents listed one health impact, 339 listed two health impacts, and 370 named three or more health impacts.

**Fig. 3:**
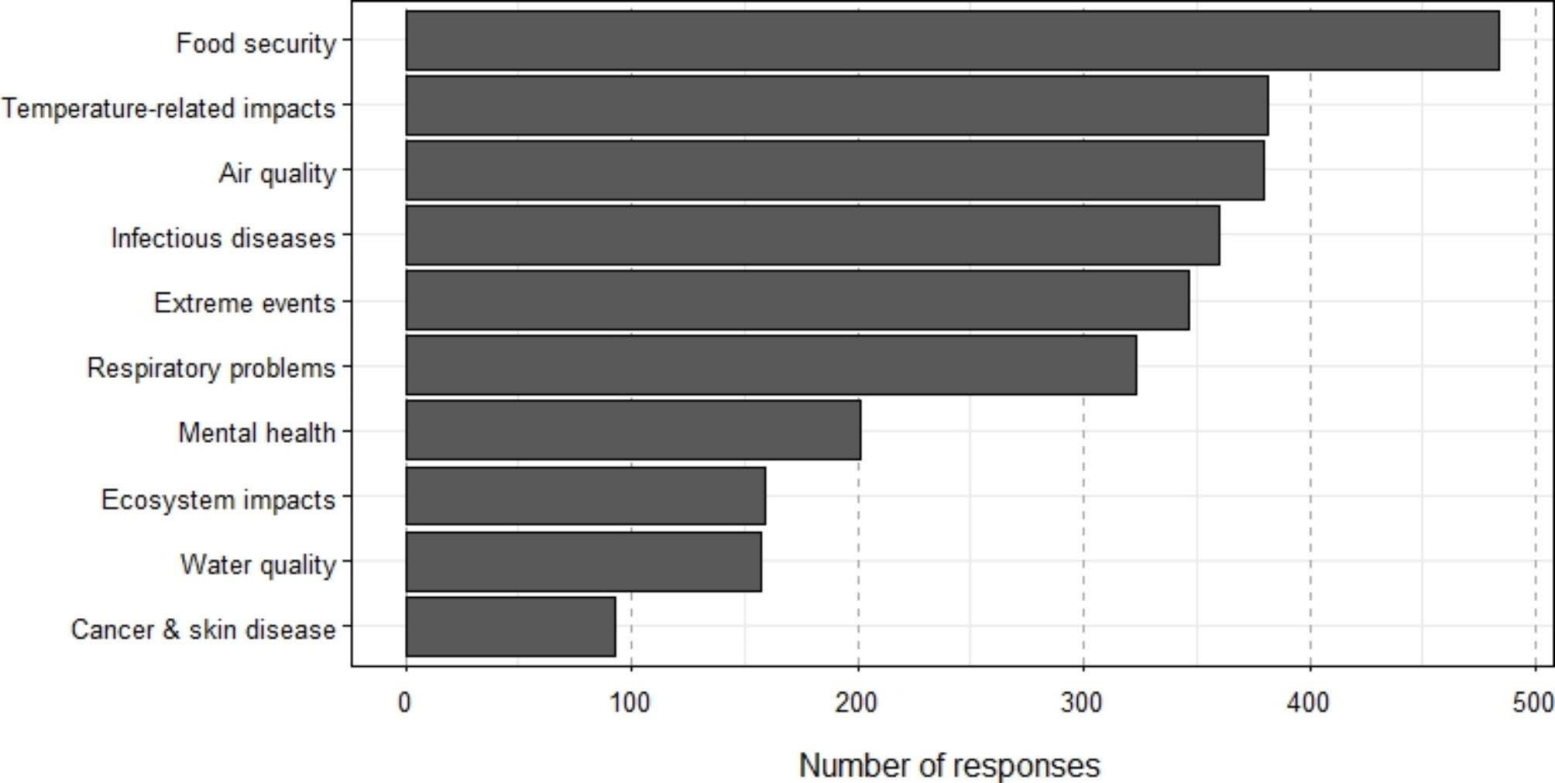
Frequencies of the ten most common health impacts identified by respondents in response to an open-ended question by climate opinion class (indirect answers such as ‘vulnerable groups’ and ‘economic losses’ are not included)

Open-ended answers were coded and 21 health impacts were identified (full list of codes and definitions in Table S2). These included conventional health impacts commonly found in the literature such as air quality and infectious diseases, as well as other more broadly interpreted impacts on health and well-being, such as impacts on economics or ecosystems. The most common climate health impacts identified were: food security and agriculture (22.3%), air quality (17.6%), temperature related morbidity and mortality (17.6%), infectious diseases (16.7%), extreme events and weather-related natural hazards (16.0%), and respiratory problems (15.0%) (Fig. 3). Additionally, some answers not related to health impacts on climate change were common, including which groups are most vulnerable to health impacts as well as the economic impacts of climate change. There were some regional differences in the frequencies of health impacts listed. Accidents and injuries were highest in the North; respiratory problems and temperature-related morbidity and mortality were highest in Central and lowest in the North; infectious diseases were higher in North and Central; and mental health was highest in the North and West.

### Water- and food-related impacts are of high concern, among other climate health impacts

**Fig. 4:**
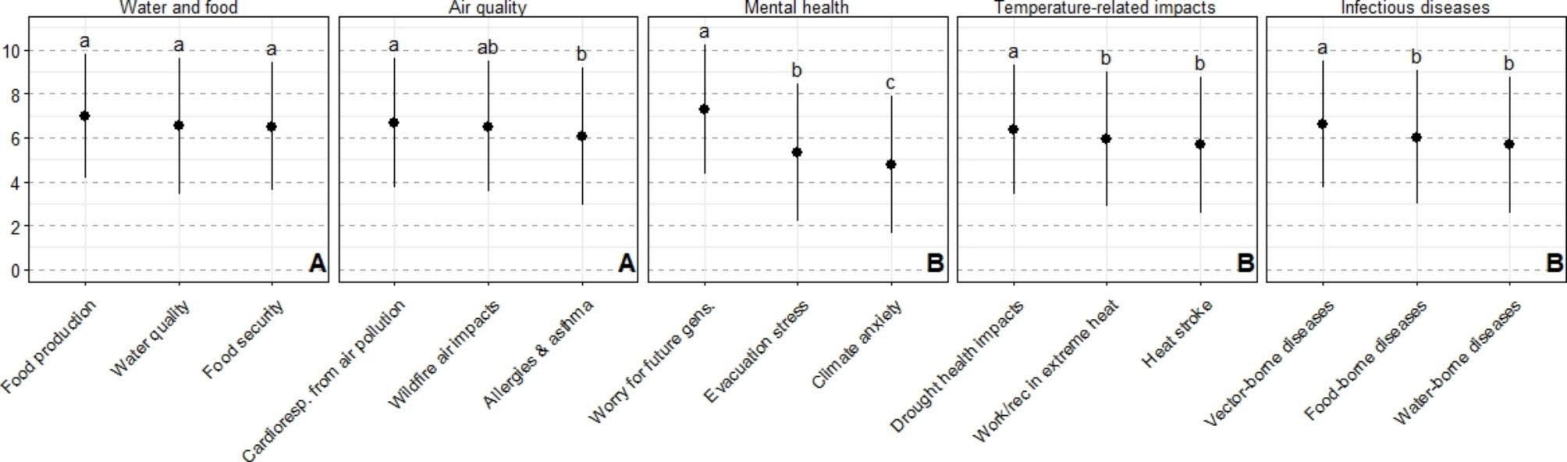
Concern about specific health-related impacts of climate change. Uppercase letters indicate differences among broad categories of health impacts; lowercase letters indicate differences among specific health impacts within a category

Respondents were empirically surveyed about their level of concern for 15 specific climate change health impacts. These 15 impacts were grouped into five categories: water- and food-related impacts, mental health, infectious diseases, temperature-related impacts, and air quality. There was significantly higher concern about impacts on water- and food-related impacts and air quality impacts, compared with the other three categories of health impacts (mental health, temperature-related impacts and infectious diseases) (Fig. 4). Within categories, there were differences among specific health impacts; notably within the mental health category, worry for future generations was significantly higher compared with stress from evacuation, which was significantly higher than climate anxiety.

**Fig. 5:**
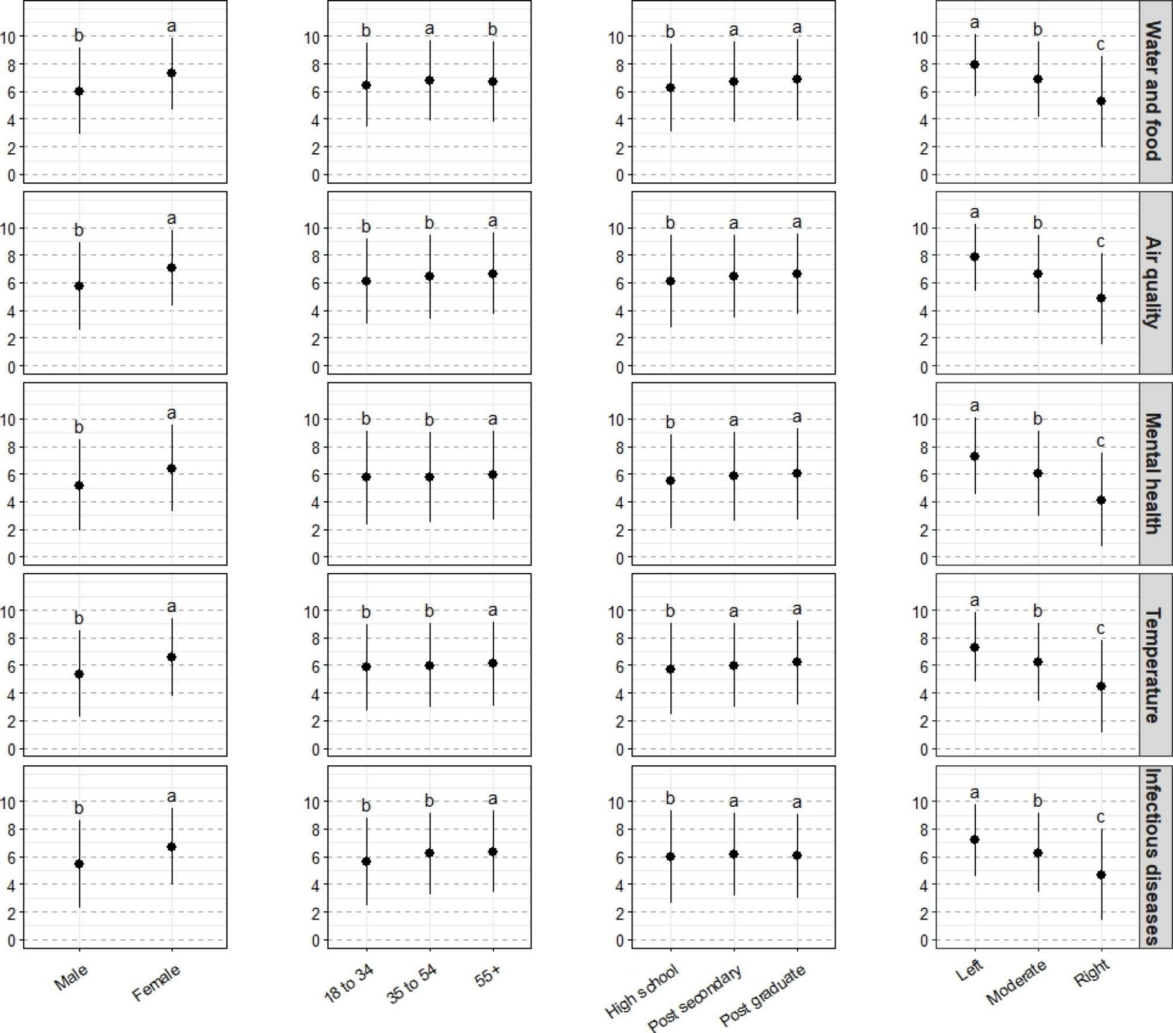
Concern about specific health-related impacts of climate change across four sociodemographic variables. Lowercase letters indicate differences among sociodemographic groups

Within the five broad categories, there were consistent differences across socio-demographic groups, and only a significant interaction in the case of age (Table S3). Women were significantly more concerned than men about all of the health-related impacts; respondents with a high school level of education were significantly less concerned about all health-related impacts compared with respondents with more education; and respondents on the political left were more concerned with those in the political centre, who were more concerned than those on the political right (Fig. 5; Table S3).

## Discussion

The goal of this study was to investigate how people across Canada understand and perceive the link between climate change and health. The study provides an understanding of public perceptions on these intersecting issues, at a time of complex and compounding public health challenges with the COVID-19 pandemic and worsening climate health impacts. The survey was conducted in the winter of 2020, and so provides a clear picture of opinions on climate and health that can be used as a benchmark to compare with other surveys both in different jurisdictions and at different times. These results offer insight that can be useful for public engagement and communications, which will allow for targeted interventions that are mindful of pre-existing climate concerns and how this affects attendant perceptions and behaviours associated with health impacts.

### Concern about health impacts of climate change

Overall, the results find that levels of concern about health impacts are similar to levels of concern about biophysical, economic, and national security impacts of climate change among people in Canada. Water- and food-related impacts are of highest concern across classes and most common in response to the open-ended question. This differs from a previous Canadian survey in which air quality impacts were the most commonly named in response to an open-ended question [15]. Somewhat surprisingly, given recent discussion on these matters (e.g. [38]), mental health concerns ranked the lowest along with temperature-related impacts and infectious diseases, although there was significantly higher “worry about future generations,” compared with “stress from evacuation during extreme weather events,” and both were significantly higher than “personal climate anxiety.” The low concern about mental health impacts is interesting considering that climate change poses a serious threat to mental health in Canada [39], with some projecting mental health impacts to be among the costliest of all health impacts [40]. At the same time, mental health has been underrepresented in research on climate change and health historically [41, 42] and a recent survey found that only 44% of Canadian public health organizations report working on climate-related mental health risks [43]. This suggests that perhaps less information has been mobilized to the public regarding this pressing health issue and its linkage with climate change. Low concern about personal climate anxiety may also be due in part to the lack of widespread understanding and a common operational definition of the term [44, 45].

On the whole, these results illustrate the well-documented phenomenon of psychological distancing, which describes how people are more likely to believe the worst impacts of climate change are far away temporally, socially, and geographically [31, 46], and thus more likely to be concerned for the future generations than their own personal well-being. These beliefs have also been documented in southern Ontario, where people were more likely to describe impacts on their children or grandchildren than on themselves [16]. Even when the impact is local, such as the increased risk of Lyme disease due to changing temperatures in southern Manitoba, members of the public can perceive the risk as distant from themselves [17]. Recent research in the US shows that the spatial and temporal distribution of climate-related health impacts are far more immediate, localized, and costly than currently understood [47], which speaks to the need to carefully consider risk communications that take into account psychological distancing that may actually run counter to the best available climate and health sciences.

In some cases, concern about the health impacts of climate change parallels indicators of vulnerability across sociodemographic characteristics. Interestingly, older people (55+), and women consistently have higher concern regarding health impacts. Given that income, age, and gender are indicators of vulnerability to climate impacts generally [48], it is increasingly clear that those who are most affected by climate change are also more likely to be concerned about the impacts, and speaks to the larger health equity issues climate change poses for Canadians. These results are consistent with previous research in other parts of the world which has found a similar relationship between indicators of vulnerability and risk perception on climate change (e.g. [49–51]).

### Consideration and affective assessment of health impacts

Respondents reported considerable prior consideration of climate change health impacts, with just over half of Canadians responding high or very high on the question of how much they had thought about the connection between climate change and health, and only one fifth of respondents answered low or very low. By comparison, research in the US in 2014 found that only a third of Americans had thought about the health impacts of climate change “a great deal” or a “moderate amount” [4], while in 2020 significantly more Americans thought that climate change health effects would become more common [52]. While a substantial number of Canadians report having thought about the link between climate change and health, the results show that this consideration is not evenly distributed across the population. People with lower educational attainment and towards the right end of the political spectrum are significantly less likely to have thought about the link between climate change and health. Though perhaps unsurprising, this correlation between sociodemographics and the climate-health link indicates that there is a significant gap in awareness for half of the population, perhaps in part due to a lack of exposure to information on health impacts. At a global level, despite increasing public and scientific work on climate change and health [53], “in absolute terms, climate change continues to be framed in ways that pay little attention to its health dimensions” [54]. Public health agencies and units are poised as effective messengers on climate change health risks [4, 55, 56], and in some jurisdictions are mandated to communicate this information, such as the Canadian province of Ontario [57]. However, a recent survey of Canadian public health organizations found that only 48% of organizations had engaged in climate change and health education and outreach with the public, and only 36% had undertaken education or training on climate change and health risks and adaptation among staff or professionals [43]. Further health-focused climate communications campaigns (e.g. [58]) are needed to target those less engaged on climate change if we hope to reach widespread understanding of and participation in adaptive health behaviours, and the results of this Canada-wide study are helpful in this regard.

The results also found that the majority of respondents perceive climate change as harmful to human health, or are inclined to answer as such when prompted, despite approximately half of respondents not having thought a substantial amount about health impacts in particular. Similarly, a 2015 survey of Americans found that while only 10% of people had previously thought about climate change and health “a great deal,” 31% responded that climate change is “very bad” for health [4]. As Maibach et al. [4] suggest, this discrepancy could be in part due to people making inferences or momentary judgements about the negative impacts of climate change on health due to their general understanding of the issue, the context of the survey, and/or their suppositions about what the “right” answer is.

### Unprompted knowledge of health impacts

More than half of Canadians can name one or more health impacts of climate change when unprompted, with the most common impacts related to food security and agriculture, air quality, temperature related morbidity and mortality, and extreme events. Answers to open-ended questions such as this are likely more realistic representations of respondents’ true understandings of climate change and health as compared with close-ended questions that may prompt certain responses [4, 59]. Results here show a slight decline compared to previous surveys which found 69% of climate change believers in 2017 and 63% of Canadians in 2008 were able to identify one or more health impacts of climate change in response to an open-ended question [13, 15]. On the other hand, the results are significantly higher than a survey of US adults in 2015 which found that only 27% of respondents could name at least one health impact of climate change unprompted. The different coding schemes used to classify open-ended responses in these different surveys may be responsible for some of the variation in results. In general, however, the results here suggest that there has not been a notable increase in public knowledge on the health impacts of climate change over the past decade.

### Opportunities and challenges for health framing within climate communications

Engaging the public with focused, effective, and audience-specific communications on health-related climate issues has several potential benefits. The public health system can inform the public about health-related risks so that steps can be taken to prevent harm and to reduce risks [60]. In addition, communicating the health-related risks may be an effective way of increasing engagement in climate change issues [61]. The gap between awareness and action is well documented in the literature, and remains a major challenge for communicators and climate advocates [30, 62–64]. Some recent literature points to a health framing as a potentially effective way to engage people across political and ideological divides [20, 21, 65], possibly because of a ‘ceiling effect’ where those on the left end on the political spectrum are already very concerned about the impacts of climate change [33]. However, our results found relatively low concern regarding climate health impacts among people whose political beliefs fall on the right end of the spectrum and suggests this may not hold true for this audience in Canada. In other studies, testing climate health framing, one found that public health framing in climate communications was effective at a local scale, but “backfired” for discussing impacts at a distance, and increased polarization across the climate concern spectrum [24]. Another study in Manitoba looking at climate change and Lyme disease similarly found a lack of resonance of climate health impacts with more sceptical audiences and suggested that health messages might be ‘strategically decoupled’ to best engage these audiences depending on the goal of the communicator [17, 66]. Results here suggest that for those less concerned about climate change, discussing the health impacts of climate change may not be more effective for engaging them than other climate change frames such as the economy. On the other hand, for communicating with those who are concerned about climate change, including those with higher educational attainment, health frames may be particularly beneficial, as this group is more concerned about impacts on health than impacts on national security and the economy. A summary of key findings and implications for health and climate communications are found in Table 2. Specific written and visual materials with health and non-health related frames were tested in the latter part of this survey study and the results will be published in a subsequent paper.

**Table 2:** Key findings and implications for health and climate communications

*Key findings of public perceptions*	*Implications for health and climate communications*
When prompted, most Canadians perceive climate change as harmful to human health.	- There is awareness of the health harms of climate change, which suggests that a public health framing in climate communications may resonate to some extent with Canadians - The majority of people are likely open to information about the health risks of climate change, given that they accept that it is harmful.
People towards the left on the political spectrum and those with higher educational attainment are relatively more concerned about climate health impacts than the rest of the Canadian population.	- Variable levels of concern suggest that health framing in climate communications is likely to be more successful in reaching those already worried about climate change
Canadians who are more concerned about climate change are also more concerned about the health impacts of climate change.	- Messages about health impacts of climate change which are of lower concern (e.g. mental health, infectious diseases) may not resonate with certain audiences. This may impact the effectiveness of a health framing of climate risks.
Canadians have different levels of concern about specific health impacts, with highest concern for future generations, food and water security, and respiratory impacts from air quality, and lowest for mental health and heat stroke impacts.	- Communicators seeking to operationalize targeted public health framings in climate change communications may choose to focus on specific health impacts of high or low concern, depending on their objectives. - Climate communicators hoping to reach the largest audiences with messages that resonate might use the areas of highest concern in their messaging.

### Limitations and areas for future research

A significant limitation of the study was the sample size from Indigenous and racialized communities which was not sufficient to allow us to analyze results by ethnicity. This is an important limitation, given that racialized communities are disproportionately impacted by climate change now and into the future, including the health impacts of climate change, due to systemic marginalization and racism [53, 67]. In this way, the survey overlooked a large and potentially significantly at-risk portion of the population. Sufficient sample representation for comparison by race is also important considering previous research in the United States which has found that People of Colour are more often concerned about climate change impacts than white people [68]. Future surveys should ensure sufficient representation from Indigenous and racialized communities through more targeted recruitment and sampling, in order for the results to be applicable and useful to these communities.

Finally, the survey is limited in its scope given a finite number of variables that could be explored and the limitations inherent with measuring evaluative judgments. As Maibach et al. [4] explains, “evaluative judgments are not necessarily comprehensive representations of an individual’s “true” attitudes, but rather are based on momentarily accessible, salient information”. The context of the questions within the survey focused on climate change could have influenced participants’ responses. Additionally, the measures here (i.e. concern, awareness, affective assessment, and unprompted knowledge) do not necessarily fully capture issue value or importance. It is possible that people are not overly concerned about the climate health impacts presented, yet they do significantly value health, which may mean that we are underestimating the effectiveness of a health frame. The conclusions drawn with respect to applications in communications framings are limited given that these results just deal with perceptions and do not directly test health frames in communications materials. Test of health framing materials was done in a subsequent part of the survey and will be published in a future article.

Future research should seek further resolution of climate health perceptions and knowledge in Canada and the efficacy of health framing between demographics, geographic regions, and locally germane climate impacts. Studies testing the effects of specific climate and health communication campaigns on uptake of adaptive behaviours and support for policy change are also needed, as an alternative measure of the efficacy of these frames, and would contribute to the evolving literature on climate/health indicators [53]. Additionally, further exploration into the relationship between people’s personal health attitudes, experiences of climate impacts, and perceptions of climate health risks may shed more light on what informs and underlies the different perceptions found here.

## Conclusion

This research has investigated public perceptions of the health impacts of climate change across Canada though a nationally-representative online survey that was carried out in the winter of 2020 at the height of the COVID-19 pandemic. We find that awareness, knowledge, and concern about climate health impacts is high across the Canadian population, especially those with higher educational attainment and who are in the centre or left of the political spectrum. This study helps to clarify the different climate concern classes across Canada, especially as they relate to health impacts, and provides a more nuanced understanding of national risk perceptions and opportunities to communicate with these various audiences. Health impacts related to food and water, air quality, and worry about future generations were of highest concern, while heat and mental health impacts were of lower concern. Given the extreme heat, wildfires and evacuations that ravaged Canada in the summer of 2021, this study sets a crucial foundation for considering how climate impacts affect perceptions of health risks moving forward. Results from this survey can inform areas of education and communications on health and climate change – of use to public health practitioners and communicators, as well as climate change communicators and researchers. Given the COVID-19 pandemic, there is greater awareness regarding health issues and thus greater potential for strengthening national understanding regarding the relationship between climate change and public health. This study suggests that there is significant work ahead – and thus opportunity – within the Canadian context to translate climate and health linkages into citizen-level understanding that will meaningfully support the societal-wide climate action that is necessary for a healthy future.

## Electronic supplementary material

Below is the link to the electronic supplementary material.

Supplementary Material 1

## Data Availability

The data that support the findings of this study are available from The University of Winnipeg but restrictions apply to the availability of these data, as they will be used in further publications. Requests for the data can be made to the corresponding author.
