# Supplementary material for: Perceptions of the health impacts of climate change among Canadians
Source: BMC Public Health. 2023 Jan 31;23:212. doi: 10.1186/s12889-023-15105-z (PMC9887551; doi:10.1186/s12889-023-15105-z)
Supplement: Supplementary file 1 — Supplementary Material 1 [file 12889_2023_15105_MOESM1_ESM.docx]

**Supplementary Information**

**Supplementary Information 1 (S1). Survey Instrument**

**Introduction**

Thank you in advance for your participation in the following survey, conducted by Nanos Research on behalf of the University of Winnipeg’s Prairie Climate Centre. The survey is will take approximately 10-15 minutes to complete. All the views and information you share with Nanos Research will be confidential and protected in accordance with Canada’s privacy laws. By completing this survey, you consent to your anonymous answers being used to develop research products which may be shared online, at conferences, and/or in academic publications. Your participation is voluntary and you may withdraw from the survey at any point prior to its completion. This project has been registered with the Canada Research and Insights Council and approved by the University of Winnipeg University Human Research Ethics Board. If you have any concerns about this survey you can send an email to ethics@uwinnipeg.ca or jhenwood@nanosresearch.com.

A. Are you 18 years of age or older?

🞎 Yes 🞎 No (*Terminate – not qualified)*

1. Do you or does anyone in your immediate family, work in any of the following occupations?

🞎 Market research firm (*Terminate – not qualified)*

🞎 TV, radio or news media (*Terminate – not qualified)*

🞎 Advertising company (*Terminate – not qualified)*

**Section 1**

1. In which province or territory do you live?

British Columbia 1

Alberta 2

Saskatchewan 3

Manitoba 4

Ontario 5

Quebec 6

New Brunswick 7

Nova Scotia 8

Prince Edward Island 9

Newfoundland and Labrador 10

Northwest Territories 11

Yukon 12

Nunavut 13

I prefer not to answer 99

How concerned or not concerned are you about the following issues currently where 0 is not at all concerned and 10 is very concerned? [RANDOMIZE]

1. The economy
2. COVID pandemic
3. Race relations
4. Climate change
5. National security
6. Food security
7. Foreign affairs
8. Habitat and species decline
9. Cyber attacks

_________ Rating

Unsure 77

1. There has been a lot of discussion lately about the issue of climate change or global warming. How certain are you that climate change is or is not happening, where 0 is extremely sure it’s not happening and 10 being extremely sure it is happening?

_________ Rating

1. Do you believe that climate change is... [ROTATE]

Caused mostly by human activity 1

Caused mostly by natural changes in the environment 2

None of the above because climate change isn’t happening 3

Other (Specify) 20

Don’t know 77

On a scale of 0 to 10 where 0 is having no impact and 10 is have a significant impact, how much do you think climate change will impact the following groups [RANDOMIZE]

1. You and your immediate family
2. Your community
3. Other people in Canada
4. People in other parts of the world outside of Canada
5. Future generations of people

_________ Rating

Unsure 77

1. Have you ever personally experienced any of the following impacts? (check all that apply) [RANDOMIZE]

Forest fire 1

Flooding 2

Drought 3

Heat wave 4

Coastal storm surge 5

Shortened winter ice season 6

Climate-related infectious disease 7

Other climate-related impact: 8

None 9

I don’t know 77

**Section 2**

How concerned or not concerned are you about the following climate risks/impacts where 0 is not at all concerned and 10 is very concerned? [RANDOMIZE]

1. Canadian national security being compromised by climate change destabilizing things in other countries
2. Impacts of more climate refugees moving to Canada on the security of your personal life presently
3. Climate change accelerating conflict and affecting the security of you and your family in the future
4. Increased migration of peoples globally in the future that put pressure on Canada’s borders

_________ Rating

Unsure 77

How concerned or not concerned are you about the following climate risks/impacts where 0 is not at all concerned and 10 is very concerned? [RANDOMIZE]

1. Current financial costs to you and your family due to climate change
2. Increased societal risk of an economic crash as climate change unfolds
3. Current increased societal costs for goods and services due to climate change affecting various industries
4. Future costs to you personally for additional insurance to address and safeguard against climate impacts moving forward

_________ Rating

Unsure 77

How concerned or not concerned are you about the following climate risks/impacts where 0 is not at all concerned and 10 is very concerned? [RANDOMIZE]

1. Current threats to your health and well-being because of climate impacts
2. Increased health risks to Canadians in the future under climate change
3. Increased number of very hot days and associated heat waves in the future affecting your health
4. Impacts to the health future generations

_________ Rating

Unsure 77

How concerned or not concerned are you about the following climate risks/impacts where 0 is not at all concerned and 10 is very concerned? [RANDOMIZE]

1. Increased personal risk of extreme events such as storms and flooding in the short term
2. Climate change increasing forest fires in the future affecting the safety of your family
3. Climate change currently affecting Canada’s ecosystems, parks, and other natural features of the landscape
4. Coastal sea level rise and erosion affecting Canadians in the future

_________ Rating

Unsure 77

**Section 3**

1. Before taking this survey, how much had you thought about how climate change might affect people’s health on a scale from 0 to 10, where 0 is hadn’t thought about it at all and 10 is thought about it a great deal?

_________ Rating

Unsure 77

1. How would you rate the impact of climate change on people’s health on a scale of 0 to 10 where 0 is very good for people’s health and 10 is very bad for people’s health?

_________ Rating

Unsure 77

1. In what ways, if any, do you think climate change will affect the health of Canadians? [OPEN ENDED]

**Section 4**

How concerned are you about the following health risks/impacts from climate change where 0 is not concerned at all and 10 is very concerned? [RANDOMIZE]

1. Heat stroke
2. Water quality and availability
3. Work and recreation outside during extreme heat
4. Personal climate anxiety
5. Diseases spread in water, such as gastrointestinal illnesses
6. Cardio respiratory problems caused by manmade air pollution
7. Asthma and allergies
8. Stress from evacuation during extreme weather events

How concerned are you about the following health risks/impacts from climate change where 0 is not concerned at all and 10 is very concerned? [RANDOMIZE]

1. Wildfire smoke causing respiratory problems
2. Health impacts of increased drought due to increasing heat
3. Food security
4. Diseases spread by insects, such as West Nile Virus or Lyme Disease
5. Agricultural food production
6. Worry about wellbeing of future generations
7. Diseases spread in food, such as E.coli

_________ Rating

Unsure 77

**Section 5**

**Stream A – Climate (Half the Sample)**

[SHOW TEXT]

Some of the projected and current impacts of climate change in Canada include:

- Melting permafrost and loss of cold in the north
- Coastal sea level rise and flooding
- Increased activity of pest and invasive species
- More variable and more extreme weather, with increased risk of weather-related catastrophes such as droughts and floods
- Hotter summers, increasing risks of drought and forest fires

Some examples of actions that can be taken to reduce climate change impacts are:

- Cutting back on emissions by replacing fossil fuels with renewable energy where possible and retrofitting buildings can help use less energy and resources
- Walking, biking, or taking public transit instead of driving can also reduce emissions.
- Creating more green spaces can reduce extreme heat and help manage flooding

Thinking of the information above, on a scale from 0 to 10, where 0 is completely disagree and 10 is completely agree, please rate your agreement with the following descriptions of the information [RANDOMIZE]

1. Interesting
2. Useful
3. Does apply to me
4. Worth sharing with others
5. Credible
6. Familiar
7. Easy to understand

_________ Rating

Unsure 77

Thinking of the information above, please rate the extent that you feel the following emotions on a scale from 0 to 10, where 0 is not at all and 10 is a great deal? [RANDOMIZE]

1. Worry
2. Helplessness
3. Anger
4. Hope
5. Motivation

_________ Rating

Unsure 77

1. How much did this information change your level of concern about climate change prior to engaging with this survey, on a scale from 0 to 10 where 0 is a lot less concerned and 10 is a lot more concerned?

_________ Rating

Unsure 77

[SHOW MAP AND TEXT]

This map shows the number of very hot days in an average summer (e.g. days when the temperature rises to at least 30C) in the future under climate change, if emissions continue to increase at present rates. When temperatures are very hot, there is a higher risk of droughts and wildfires.

Thinking of the information above, on a scale from 0 to 10, where 0 is completely disagree and 10 is completely agree, please rate your agreement with the following descriptions of the information [RANDOMIZE]

1. Interesting
2. Useful
3. Does apply to me
4. Worth sharing with others
5. Credible
6. Familiar
7. Easy to understand

_________ Rating

Unsure 77

Thinking of the information above, please rate the extent that you feel the following emotions on a scale from 0 to 10, where 0 is not at all and 10 is a great deal? [RANDOMIZE]

1. Worry
2. Helplessness
3. Anger
4. Hope
5. Motivation

_________ Rating

Unsure 77

1. How much did this information change your level of concern about climate change prior to engaging with this survey, on a scale from 0 to 10 where 0 is a lot less concerned and 10 is a lot more concerned?

_________ Rating

Unsure 77

1. Did you find this map had more or less of an impact compared to text alone on you personally where 0 is much less of an impact and 10 is much more of an impact?

_________ Rating

Unsure 77

**Stream B – Health (Half the Sample)**

[SHOW TEXT]

Some of the current and projected impacts of climate change on the health of Canadians include:

- Loss of cold, which threatens travel and food security in the north
- Threats to coastal community safety and rise in water-borne diseases
- Increase of infectious and vector-borne diseases
- More extreme events affecting communities’ physical and mental health
- More heat-related illness, such as fainting, heat exhaustion, and heat stroke

Some examples of actions that can be taken to reduce the health effects of climate change:

- Cutting back on emissions by replacing fossil fuels with renewable energy where possible will keep people safer from impacts such as extreme weather events, wildfires, and sea level rise.
- Walking, biking, or taking public transit instead of driving can increase physical and mental health while also reducing emissions.
- Creating more green spaces can protect people from heat waves and flooding, while also improving physical activity and mental health

Thinking of the information above, on a scale from 0 to 10, where 0 is completely disagree and 10 is completely agree, please rate your agreement with the following descriptions of the information [RANDOMIZE]

1. Interesting
2. Useful
3. Does apply to me
4. Worth sharing with others
5. Credible
6. Familiar
7. Easy to understand

_________ Rating

Unsure 77

Thinking of the information above, please rate the extent that you feel the following emotions on a scale from 0 to 10, where 0 is not at all and 10 is a great deal? [RANDOMIZE]

1. Worry
2. Helplessness
3. Anger
4. Hope
5. Motivation

_________ Rating

Unsure 77

1. How much did this information change your level of concern about climate change prior to engaging with this survey, on a scale from 0 to 10 where 0 is a lot less concerned and 10 is a lot more concerned?

_________ Rating

Unsure 77

[SHOW MAP AND TEXT]

**[TEXT]**

This map shows the number of very hot days in an average summer (e.g. days when the temperature rises to at least 30C) in the future under climate change, if emissions continue to increase at present rates. When temperatures are very hot, people are much more likely to suffer from health impacts, such as heat exhaustion and heat stroke, and outdoor activities become dangerous or impossible.

Thinking of the information above, on a scale from 0 to 10, where 0 is completely disagree and 10 is completely agree, please rate your agreement with the following descriptions of the information [RANDOMIZE]

1. Interesting
2. Useful
3. Does apply to me
4. Worth sharing with others
5. Credible
6. Familiar
7. Easy to understand

_________ Rating

Unsure 77

Thinking of the information above, please rate the extent that you feel the following emotions on a scale from 0 to 10, where 0 is not at all and 10 is a great deal? [RANDOMIZE]

1. Worry
2. Helplessness
3. Anger
4. Hope
5. Motivation

_________ Rating

Unsure 77

1. How much did this information change your level of concern about climate change prior to engaging with this survey, on a scale from 0 to 10 where 0 is a lot less concerned and 10 is a lot more concerned?

_________ Rating

Unsure 77

1. Did you find this map had more or less of an impact compared to text alone on you personally where 0 is much less of an impact and 10 is much more of an impact?

_________ Rating

Unsure 77

**Section 6**

Our last few questions will help us group responses.

1. What is your gender? [OPEN]
2. What year were you born??

I prefer not to answer 99

1. What is your race/ethnicity?

White 1

Black 2

Indigenous 3

Other 20

No response/refused 99

1. What size of community do you live in?

Less than 1,000 people 1

1,000-10,000 people 2

10,000-100,000 people 3

More than100,000 people 4

No response/refused 99

1. What is the highest level of education you have completed?

Elementary school 1

Some high school 2

Completed high school 3

Some community college/technical college/CEGEP 4

Completed community college/technical college/CEGEP 5

Some university 6

Completed university 7

Post-graduate degree 8

No schooling 9

No Response/Refused 99

1. What is your household income for the year?

Less than $40,000 1

$40,000 up to $75,000 2

$75,000 up to $100,000 04 3

$100,000 up to $150,000 05 4

$150,000 and over 5

DK/NA 99

1. What are the first 5 digits of your postal code?
2. In politics people sometimes talk of left and right. In terms of your political views, where would you place yourself on the scale below where 0 means left and 10 means right? _______
3. Do you practice your faith:

Every day 1

Once a week 2

Once a month 3

A couple times a year 4

Not at all 5

Refuse/no answer 99

1. On a scale of 0 to 10 where 0 is not at all concerned about your health and 10 is very concerned about your health, how would rate your concern or lack of concern about your personal health? (DK/NA)

**Table S1.** Codes for Open-ended Health Impacts

| Label | Code | n | Definition |
| --- | --- | --- | --- |
| FSA | Food security and agriculture | 494 | Food security impacts, such as increasing cost of food, food shortages, loss of crops due to weather changes, soil erosion, loss of aquaculture and fisheries |
| AQ | Air quality | 367 | Air quality, air pollution, smog, smoke from forest fires, greenhouse gas emissions |
| TRMM | Temperature-related morbidity and mortality | 357 | Health impacts from extreme heat and cold events |
| ID | Infectious diseases | 339 | Infectious diseases, including vector-borne diseases, food- and water-borne diseases, and other diseases, viruses, and pests. |
| EE | Extreme events and weather-related natural hazards | 330 | Extreme events, such as floods, tornadoes, snow storms, extreme weather, drought, wind storms, and forest fires |
| RI | Respiratory problems | 292 | Respiratory illnesses such as lung disease, asthma, allergies, breathing problems, COPD, pneumonia |
| VULN | Vulnerable groups, who is impacted | 235 | Who is most impacted by the health impacts of climate change, e.g. elderly, immune compromised, poor people, people with existing health challenges, future generations, people in specific regions |
| MH | Mental health and wellbeing | 193 | Mental health impacts such as worry, anxiety, well-being, stress and stress-related illnesses |
| TX | Economic impacts | 182 | Economic impacts such as job insecurity, paying more taxes (e.g. carbon tax), increased poverty, increases in expenses (e.g. insurance, medication rates), increased cost of living, transportation and home expenses |
| OT | Other | 179 |  |
| WI | Water quality and security | 173 | Impacts on oceans and freshwater, such as availability and quality of drinking water and sea level rise |
| ECO | Biophysical impacts and effects on ecosystems | 155 | Environmental changes (e.g. changing coastlines, loss of forests and biodiversity melting permafrost, melting of ice) |
| CAN | Cancers and skin disease | 95 | Cancers such as skin cancer and melanoma |
| PD | Population displacement, migration | 56 | Impacts of climate displacement, overpopulation, migration |
| CI | Cardiovascular illness | 47 | Cardiovascular impacts such as heart attacks, strokes, blood pressure |
| PHS | Impacts on public health system | 43 | Strains on health care system, more people needing health care, overwhelmed hospitals |
| PC | Positive consequences | 41 | Positive impacts of climate change on health, such as people spending more time outside and longer growing seasons. |
| HI | Housing insecurity | 38 | Lost or destroyed housing |
| AI | Accidents and Injuries | 37 | Accidents or injuries, such as slipping on ice, injuries from natural disasters, drowning |
| CON | Conflicts and crisis | 22 | Increased conflicts such as war and political crises |
| OCC | Occupational health risks | 7 | Increased health risks in working conditions due to climate impacts (e.g. extreme heat for those working outside) |

**Table S2. Two-way ANOVA results among climate impacts and sociodemographic groups.**

|  | df | F value | p value |
| --- | --- | --- | --- |
| Gender | 1 | 24.5 | <0.001 |
| Climate Impact | 3 | 804.69 | <0.001 |
| Gender*Climate Impact | 3 | 29.88 | <0.001 |
|  |  |  |  |
| Age | 2 | 26.587 | <0.001 |
| Climate Impact | 3 | 2488 | <0.001 |
| Age*Climate Impact | 6 | 4.523 | <0.001 |
|  |  |  |  |
| Education | 2 | 24.573 | <0.001 |
| Climate Impact | 3 | 29.801 | <0.001 |
| Education*Climate Impact | 6 | 5.093 | <0.001 |
|  |  |  |  |
| Politics | 2 | 21.77 | <0.001 |
| Climate Impact | 3 | 248.76 | <0.001 |
| Politics*Climate Impact | 6 | 44.67 | <0.001 |

**Table S3.** Two-way ANOVA results among types of health impacts and sociodemographic groups.

|  | df | F value | p value |
| --- | --- | --- | --- |
| Gender | 1 | 24.5 | <0.001 |
| Climate Impact | 3 | 804.69 | <0.001 |
| Gender*Climate Impact | 3 | 29.88 | <0.001 |
|  |  |  |  |
| Age | 2 | 26.587 | <0.001 |
| Climate Impact | 3 | 2488 | <0.001 |
| Age*Climate Impact | 6 | 4.523 | <0.001 |
|  |  |  |  |
| Education | 2 | 24.573 | <0.001 |
| Climate Impact | 3 | 29.801 | <0.001 |
| Education*Climate Impact | 6 | 5.093 | <0.001 |
|  |  |  |  |
| Politics | 2 | 21.77 | <0.001 |
| Climate Impact | 3 | 248.76 | <0.001 |
| Politics*Climate Impact | 6 | 44.67 | <0.001 |


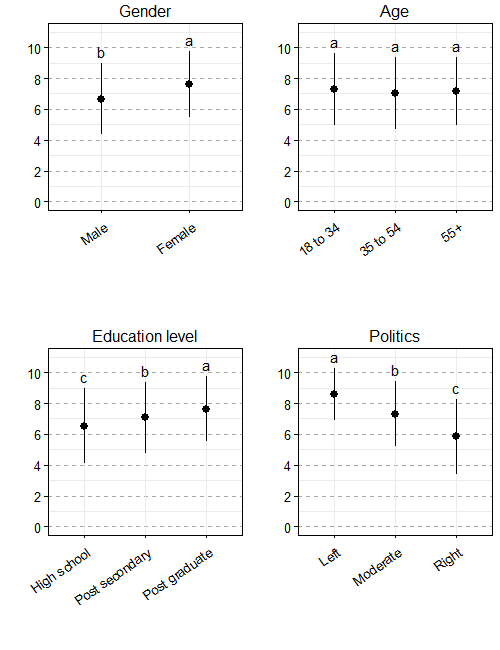


FIG S1: Opinion about the impact of climate change on health. Lowercase letters indicate statistical differences among sociodemographic groups.
